# LTR_FINDER_parallel: parallelization of LTR_FINDER enabling rapid identification of long terminal repeat retrotransposons

**DOI:** 10.1186/s13100-019-0193-0

**Published:** 2019-12-12

**Authors:** Shujun Ou, Ning Jiang

**Affiliations:** Department of Horticulture, Michigan State University, East Lansing, MI 48824 USA

**Keywords:** Genome annotation, Transposable element, LTR retrotransposon, LTR_FINDER

## Abstract

Annotation of plant genomes is still a challenging task due to the abundance of repetitive sequences, especially long terminal repeat (LTR) retrotransposons. LTR_FINDER is a widely used program for the identification of LTR retrotransposons but its application on large genomes is hindered by its single-threaded processes. Here we report an accessory program that allows parallel operation of LTR_FINDER, resulting in up to 8500X faster identification of LTR elements. It takes only 72 min to process the 14.5 Gb bread wheat (*Triticum aestivum*) genome in comparison to 1.16 years required by the original sequential version. LTR_FINDER_parallel is freely available at https://github.com/oushujun/LTR_FINDER_parallel.

## Introduction

Transposable elements (TEs) are the most prevalent components in eukaryotic genomes. Among different TE classes, long terminal repeat (LTR) retrotransposons, including endogenous retroviruses (ERVs), is one of the most repetitive TEs due to their high copy numbers and large element sizes [1]. LTR retrotransposons are found in almost all eukaryotes including plants, fungi, and animals, but are most abundant in plant genomes [2]. For example, LTR retrotransposons contribute more than 65 and 70% to the genomes of bread wheat (*Triticum aestivum*) and maize (*Zea mays*), respectively [1].

Annotation of LTR retrotransposons relies primarily on de novo approaches due to their highly diverse terminal repeats. For this purpose, many computational programs have been developed in the past two decades. LTR_FINDER is one of the most popular LTR search engines [3], and the prediction quality out-performs counterpart programs [1]. However, LTR_FINDER runs on a single thread and is prohibitively slow for large genomes with long contigs, preventing its application in those species. In this study, we applied the “divide and conquer” approach to simplify and parallel the annotation task for the original LTR_FINDER and observed an up to 8500 times speedup for analysis of known genomes.

## Methods

We hypothesized that complete sequences of highly complex genomes may contain a large number of complicated nested structures that exponentially increase the search space. To break down these complicated sequence structures, we split chromosomal sequences into relatively short segments (1 Mb) and executes LTR_FINDER in parallel. We expect the time complexity of LTR_FINDER_parallel is *O*(n). For highly complicated regions (i.e., centromeres), one segment could take a rather long time (i.e., hours). To avoid extended operation time in such regions, we used a timeout scheme (300 s) to control for the longest time a child process can run. If timeout, the 1 Mb segment is further split into 50 Kb segments to salvage LTR candidates. After processing all segments, the regional coordinates of LTR candidates are converted back to the genome-level coordinates for the convenience of downstream analyses.

LTR_FINDER_parallel is a Perl program that can be "download and run" and does not require any form of installation. We used the original LTR_FINDER as the search engine which is binary and also installation free. Based on our previous study [1], we applied the optimized parameter for LTR_FINDER (−w 2 -C -D 15000 -d 1000 -L 7000 -l 100 -p 20 -M 0.85), which identifies long terminal repeats ranging from 100 to 7000 bp with identity ≥85% and interval regions from 1 to 15 Kb. The output of LTR_FINDER_parallel is convertible to the popular LTRharvest [4] format, which is compatible to the high-accuracy post-processing filter LTR_retriever [1].

## Results

To benchmark the performance of LTR_FINDER_parallel, we selected four plant genomes with sizes varying from 120 Mb to 14.5 Gb, which are *Arabidopsis thaliana* (version TAIR10) [5], *Oryza sativa* (rice, version MSU7) [6, 7], *Zea mays* (maize, version AGPv4) [8], and *Triticum aestivum* (wheat, version CS1.0) [9], respectively. Each of the genomes was analyzed both sequentially (1 thread) and in parallel (36 threads) with wall clock time and maximum memory recorded.

Using our method, we observe 5X - 8500X increase in speed for plant genomes with varying sizes (Table 1). For the 14.5 Gb bread wheat genome, the original LTR_FINDER took 10,169 h, or 1.16 years, to complete, while the multithreading version completed in 72 min on a modern server with 36 threads, demonstrating an 8500X increase in speed (Table 1). Even we analyzed each wheat chromosome separately, the original LTR_FINDER still took 20 days on average to complete. Among the genomes we tested, the parallel version of LTR_FINDER produced slightly different numbers of LTR candidates when compared to those generated using the original version (0–2.73%; Table 1), which is likely due to the use of the dynamic task control approach for processing of heavily nested regions. By filtering out LTR candidates in the rice genome with LTR_retriever [1], we found a difference of 28 LTR elements (out of ~ 1950 filtered elements) between results from the original version and the parallel version of LTR_FINDER. Of these 28 elements, 25 of them were located at the sequence split sites. However, all of the 28 elements were represented by similar full-length copies identified from other locations in the genome, indicating little loss in terms of the coverage of final library for LTR retrotransposons. Given the substantial speed improvement (Table 1), we consider the parallel version to be a promising solution for large genomes.

**Table 1:** Benchmarking the performance of LTR_FINDER_parallel

Genome	Arabidopsis	Rice	Maize	Wheat
Version	TAIR10	MSU7	AGPv4	CS1.0
Size	119.7 Mb	374.5 Mb	2134.4 Mb	14,547.3 Mb
Original memory (1 thread^a^)	0.37 Gbyte	0.55 Gbyte	5.00 Gbyte	11.88 Gbyte^b^
Parallel memory (36 threads^a^)	0.10 Gbyte	0.12 Gbyte	0.82 Gbyte	17.67 Gbyte
Original time (1 thread)	0.58 h	2.1 h	448.5 h	10,169.3 h^b^
Parallel time (36 threads)	6.4 min	2.6 min	10.3 min	71.8 min
Speed up	5.4 X	48.5 X	2613 X	8498 X
# of LTR candidates (1 thread)	226	2851	60,165	231,043
# of LTR candidates (36 threads)	226	2834	59,658	237,352
% difference in candidate #	0.00%	0.60%	0.84%	−2.73%

^a^ Intel(R) Xeon(R) CPU E5–2660 v4 @ 2.00GHz

^b^ LTR_FINDER was run on each chromosome; the maximum memory and the total time are shown

## Data Availability

LTR_FINDER_parallel is freely available at https://github.com/oushujun/LTR_FINDER_parallel.

## Abbreviations

ERV: Endogenous retrovirus
LTR retrotransposon: Long terminal repeat retrotransposon
TE: Transposable element
